# Real-life outcomes in biotypes of psychotic disorders based on neurocognitive performance

**DOI:** 10.1007/s00406-022-01518-1

**Published:** 2022-11-22

**Authors:** Vicente Molina, Inés Fernández-Linsenbarth, María Queipo-de-Llano, María Teresa Jiménez-Aparicio, Carmen Vallecillo-Adame, Abril Aremy-Gonzaga, Celia de-Andrés-Lobo, María Recio-Barbero, Álvaro Díez, Rosa M. Beño-Ruiz-de-la-Sierra, Carmen Martín-Gómez, Javier Sanz-Fuentenebro

**Affiliations:** 1 Psychiatry Service, Clinical Hospital of Valladolid, Valladolid, Spain; 2 Psychiatry Department, School of Medicine, University of Valladolid, Av. Ramón y Cajal, 7, 47005 Valladolid, Spain; 3 Psychiatry Service, Cruces Hospital, Bilbao, Spain; 4 Psychiatry Service, Clinical Hospital of Salamanca, Salamanca, Spain; 5 Spain Psychiatry Service, Doce de Octubre Hospital, Madrid, Spain

**Keywords:** Schizophrenia, Subtypes, Bipolar, Biotypes, Neurocognition, Outcomes

## Abstract

Aiming at discerning potential biotypes within the psychotic syndrome, we have recently reported the possible existence of two clusters or biotypes across schizophrenia and bipolar disorder characterized by their cognitive performance using the Brief Assessment of Cognition in Schizophrenia (BACS) instrument and validated with independent biological and clinical indexes (Fernández-Linsenbarth et al. in Schizophr Res 229:102–111, 2021). In this previous work, the group with larger cognitive deficits (*N* = 93, including 69 chronic schizophrenia, 17 first episodes (FE) of schizophrenia and 7 bipolar disorder patients) showed smaller thalamus and hippocampus volume and hyper-synchronic electroencephalogram than the group with milder deficits (*N* = 105, including 58 chronic schizophrenia, 25 FE and 22 bipolar disorder patients). We predicted that if these biotypes indeed corresponded to different cognitive and biological substrates, their adaptation to real life would be different. To this end, in the present work we have followed up the patients’ population included in that work at 1st and 3rd years after the date of inclusion in the 2021 study and we report on the statistical comparisons of each clinical and real-life outcomes between them. The first cluster, with larger cognitive deficits and more severe biological alterations, showed during that period a decreased capacity for job tenure (1st and 3rd years), more admissions to a psychiatric ward (1st year) and a higher likelihood for quitting psychiatric follow-up (3rd year). Patients in the second cluster, with moderate cognitive deficits, were less compliant with prescribed treatment at the 3rd year. The differences in real-life outcomes may give additional external validity to that yielded by biological measurements to the described biotypes based on neurocognition.

## Introduction

The heterogeneity of psychoses poses a validity problem for current diagnostic categories and thus hampers individual prognoses based on these categories. In this context, neurocognition varies across the main domains affected in patients with schizophrenia, such as verbal and working memory, motor and performance speed, verbal fluency, social cognition and problem-solving [2]. Growing evidence has indicated that different cognitive subgroups may exist within the psychosis spectrum [1–6]. Preliminary evidence suggests that such cognitive subgroups may be associated with distinctive brain structure [1, 7, 8].

In our previous work, neurocognitive data from the Spanish version of the Brief Assessment of Cognition in Schizophrenia (BACS) [9] and the Wisconsin Card Sorting Test [10] were analyzed by a K-means cluster approach to identify clusters of patients with a trans-diagnostic approach [1]. Clinical and biological data were then used to validate them. Based on the neurocognitive profile, the analysis yielded a two-cluster solution, with a severely impaired group (Cluster 1) and a moderately impaired group (Cluster 2), integrated of 93 and 105 patients, respectively. Chronic schizophrenia patients and first episodes of schizophrenia (FE) were similarly distributed between clusters, but bipolar patients were more likely to fall into Cluster 2. No significant differences for antipsychotic doses and psychiatric hospitalizations were found between clusters. Cluster 1 patients showed higher positive and negative symptom scores, longer mean illness duration (188.8 vs. 117.3 months) and were slightly older (42.3 vs. 36.9 years) than Cluster 2 patients. Regarding the biological differences, the severely impaired group was associated with lower thalamus and hippocampus volume, prefrontal connectivity alterations assessed with DTI and basal hypersynchrony of the electroencephalogram signal. These alterations were absent in the cluster with milder cognitive deficits [1].

These differences in brain structure and function seem coherent with the reports of biologically based subgroups that may help in delimitating valid clusters within and/or across diagnostic categories [7, 11–13]. A strategy to test the validity of these clusters is comparing relevant measurements (biological and clinical) independent of those used for cluster definition [12]: if clusters are valid, differences among them should not be restricted to the criteria used in their definition. In this direction, real-life adjustment among subjects belonging to those clusters, if demonstrated, could be considered as an additional source of validity together with differential biological characteristics. Belonging to one particular biotype could predict outcomes in relevant aspects of real life in the medium term.

On the other hand, psychosocial functioning may be predicted by neurocognition in schizophrenia and bipolar disorder [14]. Indeed, neurocognition has been identified as a predictor of everyday functioning, independently of positive and negative symptoms [15–17]. In particular, it has been associated with everyday life and work skills [16, 18]. Thus, the biotype with larger neurocognitive deficits could show a poorer adaptation to real-life demands. Specifically, neurocognitive deficits such as speed performance may predict job tenure [19], so job tenure may be different in the medium term depending on the neurocognitive biotype. Testing prospectively this possibility may help in assessing the validity of the reported biotypes.

Other important aspects of everyday functioning, such as interpersonal relations, may also vary in relation to neurocognition and thus differ between biotypes. Subjective quality of life, including social contacts and work, was related to neurocognitive performance [20]**.** Different aspects of neurocognition (executive functioning, motor skills and working memory) are related to satisfaction with family and social contacts [21, 22]. Finally, clinically relevant aspects such as adherence to antipsychotic treatment also relate to cognitive performance [23] from which it can be predicted that patients with lower cognitive performance may relapse more frequently and be readmitted to psychiatric wards.

We hypothesized here that subjects in different biotypes in our previous study [1] may differ in the medium term in their clinical outcome and real-life adaptation, in job tenure and interpersonal relationships, as well as in treatment adherence and its related consequences. We expected poorer results in the biotype with larger cognitive deficit. Although a worse adaptation to real-life demands could relate to patients’ impaired cognition, the demonstration of significant differences in that respect in the group where a characteristic biological pattern has previously been shown could be relevant to the validation of such a biotype. In addition, the clinical relevance of the previously identified subgroups may be enhanced if differences in real-life outcomes were supported.

To this purpose, we analyzed the differences between patients falling into the groups, respectively, characterized by severe and mild cognitive deficits in a series of clinical and real-life measurements. These groups might be called “cognitive subtypes”, given the primary classification criteria used or instead biotypes, given the relevant biological differences found between them. Considering this, we have opted by the “‘biotype” term, since not only cognitive but also relevant biological patterns characterized them. It should be noted here that this is not a strictly longitudinal study, but a study whose main goal is the validation with clinical and functional variables of the previously identified biotypes. For that purpose, we collected data in two different time points after patients’ inclusion in the biotypes study. This nature of the study explains the inevitable dropout of some patients. Nevertheless, we believe that this analysis supports the validity of the biotypes.

## Methods

### Subjects

In this study, we made an analysis at two different time points of the 198 patients included in our former report on clusters based on cognitive performance [1] who were diagnosed either as schizophrenia or bipolar disorder according to DSM-5 criteria. They included 127 with chronic schizophrenia, 42 first episodes of schizophrenia and 29 with type I bipolar disorder. As previously mentioned, after clustering based on cognition, clinical and biological variables were used for clusters’ validation.

Exclusion criteria were the same as in the previous study on clusters: (a) intelligence quotient under 70; (b) present or past substance dependence (excluding caffeine and nicotine); (c) head trauma with loss of consciousness; and (d) neurological or mental diagnosis different to schizophrenia or bipolar disorder. The study received the corresponding Institutional Review Board approval.

### Data assessment

We collected a battery of data at baseline and at 12- and 36-month time points following their inclusion in the biotypes study. Most cases were included in the 2021 report while admitted in a day clinic following a psychotic relapse or owing to lack of response. Typical stay in this clinic is 3 months, thus the 1st and 3rd years’ time points were estimates for medium-term outcomes. Two separated time points were chosen to assess the stability of the outcomes in the subgroups defined based on cognitive and biological characteristics, allowing for a sufficient time to the manifestation of outcome that might delay a longer period than others might. Some events can occur shortly after being discharged from that clinic, such as dropping out treatments or being readmitted into a psychiatric unit. Similarly, some of the chosen outcomes can be better assessed within a longer follow-up, such as the time spent on a paid job.

At baseline, we collected the following data: presence of paid job, work status (differentiating between active or inactive, i.e., paid work or other employment possibilities such as studying or volunteering, respectively), recent admissions in psychiatric wards, independent living (i.e., living outside the nuclear family), clozapine prescription, drug or alcohol abuse, and PANSS scores. Regarding the analysis, at each time point the collected data corresponded to the last year (i.e., the first or the third year after inclusion in the biotypes study). Specifically, we collected the following data at each time point: (1) job tenure (assessed as the number of weeks with paid job during the last year); (2) treatment compliance (i.e., percent of prescriptions withdrawn from pharmacy office); (3) number of readmissions in psychiatric wards; (4) alcohol or other drug abuse (according to clinical records); (5) clozapine prescriptions (according to clinical records, representing the number of treatment-resistant cases); (6) presence of satisfactory interpersonal relations (i.e., the existence of any subjectively satisfactory friendship or romantic relationship outside the nuclear family, according to the subjects responses to direct questioning); and (7) clinical monitoring (i.e., attendance at clinical appointments at the corresponding mental health center). The later four items were operationalized as Yes or Not.

It should be noted here that access to the healthcare system in Spain is unlimited and free for all these patients, who receive their medications at no or very low cost. Thus, differences in accessibility to health care are unlikely between clusters. A large majority of psychosis cases are well known in their mental health centers, with frequent appointments with their psychiatrists, nurses and social workers. Moreover, these patients are actively followed up, i.e., if appointments are missed, they are phoned and new appointments arranged, and/or nurses visit them at home. Therefore, their clinical status and social outcomes can be ascertained through medical records and interviews with patients and/or their treating psychiatrists, without need for questionnaires to reliably assess current working or personal situations such as those included in the present report.

Here, the interviewers were psychiatrists or psychiatrists in training in the healthcare centers where the patients were being treated at each assessment. Treatment compliance, alcohol or drug abuse, readmissions in psychiatric wards, clozapine prescriptions and clinical monitoring were assessed using available registers. Job tenure and satisfactory interpersonal relations were collected through personal or telephonic interviews with patients and/or the treating psychiatrist. During the follow-up periods, patients received the treatment as usual, prescribed by their psychiatrists without any restrictions. Data collection was not possible for cases when the corresponding period of the analysis was not still complete (i.e., if the patient was included in the biotypes study less than 3 years ago), collaboration was rejected by the patient, registers were incomplete, or the case was lost to follow-up in the corresponding mental health clinic. Given the nature of the study, there was an unavoidable loss of available data in some of the assessed variables (see Table 1). Participants with at least one value in one of the assessed variables were included in further analysis.

**Table 1:** Number of cases with available data per each variable and cluster for each time point

	1st year		3rd year	
	Cluster 1	Cluster 2	Cluster 1	Cluster 2
Job tenure	35	46	31	44
Treatment compliance	51	66	22	45
Readmissions	49	71	37	66
Alcohol/other drugs abuse	52	76	41	72
Clozapine	49	70	35	64
Interpersonal relations	37	45	31	40
Clinical monitoring	48	70	39	67

### Statistical analyses

Main categorical variables are described as frequencies and percentages, while continuous ones are described using mean and standard deviation. At 1st and 3rd year points, each parameter was compared between clusters using *Χ*^*2*^ for qualitative and *t* tests for quantitative data. We also compared clusters for baseline values related to the outcome variables.

Since we planned several a priori comparisons before the experiment, each can be considered a distinct and different hypothesis, so the risk of a Type 1 error is an acceptable 0.05 [24]. Therefore, we kept the level of significance at the usual *p* < 0.05.

We assessed the significance of the differences between clusters in percent of cases lost for any of the reasons stated before. We also compared clinical severity at baseline (PANSS scores) between patients where data collection was not possible for any of the above stated reasons with those where data were available.

Finally, since Cluster 1 patients were slightly older than those in Cluster 2 [1], we planned to test using forward stepwise lineal regression if the differences in quantitative real-life outcomes between clusters could relate to illness duration. As for categorical variables with significant differences, we used a Mann–Whitney *t* test to compare age between cases in each category (for example, between cases with or without follow-up at a given time point).

## Results

### Baseline

At baseline (i.e., before inclusion), patients in Cluster 2 were working and abused drugs more frequently than those in Cluster 1, without significant differences in clozapine prescriptions, alcohol abuse, living independently, work status (i.e., active/inactive) and number of prior admissions (Table 2).

**Table 2:** Demographic characteristics and baseline status in Clusters 1 and 2 of variables related to outcomes registered for this study

	Cluster 1	Cluster 2
*N* = 164	69	95
Sex (% female)	44.93%	37.89%
Age	40.32 (11.61)	37.55 (10.90)
Diagnostic (% chronic schizophrenia)	71.01%	53.68%
Illness duration (months)	175.30 (144.114)	128.77 (124.558)*
PANSS positive	13.61 (5.44)	11.60 (4.72)*
PANSS negative	19.56 (8.60)	14.04 (6.42)*
PANSS total	64.24 (22.93)	49.21 (19.31)*
Paid job (% Yes)	11.59%	29.47%**
Work status (% Active)	33.33%	47.37%
Prior admissions (lifetime)	3.68 (4.85)	2.59 (2.61)
Independent living (% Yes)	31.88%	32.63%
Clozapine (% Yes)	15.94%	11.58%
Drug abuse (% Yes)	8.69%	21.05%*
Alcohol abuse (% Yes)	10.14%	13.68%

Data are given as a percentage of the total sample included for each cluster except for age, illness duration, PANSS, and prior admissions, given as mean (standard deviation)

*p < 0.05

**p < 0.005

### Data availability and symptom scores

At 1st year after inclusion, data on 128 participants were available (52 from Cluster 1 and 76 from Cluster 2). Differences in data availability between clusters were not significant (Cluster 1: 52 out of 69; Cluster 2: 76 out of 95; χ^2^ = 0.506, *df* = 1, *p* = 0.479). However, at 3rd year (with 113 cases available), less Cluster 1 patients were available (Cluster 1: 41 out of 69; Cluster 2: 72 out of 95; χ^*2*^ = 4.330, *df* = 1, *p* = 0.037). Table 3 shows the demographic characteristics per cluster for each time point.

Baseline total PANSS scores were higher for patients lost at follow-up at 1st year (59.0, sd 23.2 vs 48.2, sd 18.5; *t* = 2.90, *p* = 0.004). Negative PANSS scores were also higher in these patients (18.1, sd 8.8 vs 14.7 sd 7.2; *t* = 2.35, *p* = 0.02), but positive PANSS scores showed no significant differences (12.2, sd 4.9 vs 10.9 sd 3.6; *t* = 1.66, *p* = 0.09). At 3rd year, no baseline PANSS differences were found between patients with data available or not.

### Job tenure

During the 1st (*t* = 2.739, *df* = 79, *p* = 0.004) and 3rd (*t* = 2.763, *df* = 79, *p* = 0.005) years, patients in Cluster 2 had worked for a significantly longer time than those in Cluster 1 (Table 3).

**Table 3:** Demographic characteristics and outcomes at 1st and 3rd years after inclusion in the biotypes study

	Cluster 1		Cluster 2	
	1st year	3rd year	1st year	3rd year
Sex (% female)	59.72%	41.46%	55.77%	36.11%
Age	40.27 (11.82)	40.15 (12.20)	38.18 (11.28)	40.21 (10.34)
Diagnostic (% chronic schizophrenia)	90.38%	92.68%	75.00%	68.05%
Job tenure (weeks in last year)	6.63 (14.48)**	5.61 (22.28)**	20.24 (26.34)	29.93 (47.72)
Treatment compliance (% last year)	93 (15.9)	97 (10.7)*	88 (20.3)	87 (26.0)
Readmissions (mean, sd)	0.80 (1.20)*	0.97 (1.42)	0.36 (0.72)	0.66 (1.04)
Interpersonal relations (% Yes)	51.35%^#^	54.83%^#^	68.89%	75.00%
Clozapine (% Yes)	22.45%	22.85%	22.86%	21.87%
Alcohol abuse (% Yes)	18.75%	23.68%	18.3%	18.51%
Other drugs (% Yes)	9.37%	5.26%	10.20%	3.70%
Lost to follow-up	8.33%	20.58%**	2.85%	2.00%

Each of the data correspond to the immediately anterior 12-months period

^a^Mean (standard deviation) or percent in each case is indicated. Significant comparisons Cluster 1 vs. Cluster 2 are reported for 1st and 3rd years follow-up: *p < 0.05; **p < 0.005; and trend level #*p* < 0.10

### Treatment compliance

At 1st year, treatment compliance was similar between clusters (Table 3). Conversely, at 3rd year patients in Cluster 2 complied significantly worse with their prescribed treatment (*t* = 2.057, *df* = 47, *p* = 0.04).

### Readmissions

Patients in Cluster 1 were readmitted to a psychiatric ward more frequently than those in Cluster 2 during the 1st year (*t* = 2.45, *df* = 119, *p* = 0.01; Table 3). The trend was the same, but not statistically significant, during the 3rd year.

### Alcohol or another drug abuse

There were no significant differences in the proportion of cases abusing alcohol or other drugs between clusters at 1st or 3rd years (Table 3).

### Clozapine

There was no difference in the proportion of cases for whom clozapine was prescribed during the 1st and 3rd years (Table 3).

### Interpersonal relations

Patients in Cluster 2 reported more frequently than patients in Cluster 1 having satisfactory interpersonal relations out of family, although only at a trend level (1st year: χ^*2*^ = 2.62, *p* = 0.1; 3rd year: χ^*2*^ = 3.17, *p* = 0.07; Table 3).

### Clinical monitoring

At 1st year, clusters were not significantly different in continuing attendance at clinical appointments at the corresponding mental health center. However, at 3rd year a larger number of cases in Cluster 1 had been lost to follow-up (χ^*2*^ = 10.77, *df* = 2, *p* = 0.005; Table 3).

### Post hoc analyses

Using stepwise regression, we found that illness duration was unrelated to the amount of tenured job at years 1st (*R*^2^ = 0.01, *F* = 0.64, *p* = ns) and 3rd (*R*^2^ = 0.01, *F* = 0.00, *p* = ns) after inclusion in the biotypes study. Similarly, percent of treatment compliance at year 3rd (*R*^*2*^ = 0.07, *F* = 2.81, *p* = ns) and number of readmissions at year 1st (*R*^2^ = 0.01, *F* = 0.87, *p* = ns) were also unrelated to illness duration.

As for categorical variables with significant differences between clusters, illness duration did not differ between patients with or without clinical monitoring at year 3rd (*U* = 251, *z* = − 1.38, *p* = ns) and with or without significant relationships at year 1st (*U* = 358, *z* = − 0.53, *p* = ns) or 3rd (*U* = 212, *z* = − 1.10, *p* = ns).

## Discussion

In this study, our aim was to contribute to the validation of the clusters identified on the basis of cognitive performance and biological data [1] using real-life and clinical outcomes in the medium term. According to the present results, these data were different between those clusters, which lends support to its validity. We consider of interest that both clusters in our previous report included schizophrenia (chronic and FE) and bipolar patients. Ecological differences between clusters, such as job tenure and interpersonal relations, revealed more difficulties in real-life functioning in Cluster 1 (i.e., the one with higher cognitive deficits), and the higher number of readmissions in this cluster suggests greater clinical severity.

According to our data, patients in Cluster 1 differed from Cluster 2 patients more obviously at year 3 than at year 1 time points. During the 3rd year, they not only worked less weeks (which was also evident at 1st year), but also had more frequently dropped follow-up and complied worse with the prescribed treatment. This would suggest that real-life adaptation may be hampered from the beginning in Cluster 1 and such adaptation is further compounded by more frequent treatment dropout. There are multiple factors that may influence real-life outcomes in psychotic patients beyond the proposed biotypes, such as living environment, parental socioeconomic status and accessibility to health care or duration of illness, among others, and its role should be investigated. Nevertheless, some of these factors such as accessibility to health care are unlikely to play a significant role in the differences here reported between clusters since it is equal and free for all patients in Spain. Moreover, the possible role of such factors would not discard a significant influence for biotypes on medium-term outcome. Indeed, the differences in job tenure were not explained by age or illness duration differences alone, despite the differences in these variables between clusters. This suggest that cognitive profiles of the clusters may contribute per se to outcome. Of course, negative symptoms may also play a role in that outcome, but since these symptoms were more severe in the cluster with higher cognitive deficits, its influence may be another aspect of the same phenomenon.

Previous data tend to support a relation between baseline cognitive performance and functional outcome [19, 25], but other studies did not support such relation [26]. This discrepancy may be due in part to an overlapping of patients from diverse biotypes: If the relation between cognition and functional outcome is examined in a group with a large proportion of “Cluster 2” patients, differences in functional outcomes in comparison with other clinical or healthy groups may be less evident. Such differences could be much stronger, however, in a group with a larger proportion of “Cluster 1” cases as compared to other clinical groups or healthy controls. Beyond this, the assessment of performance-based measures of functional capacity in addition may have also a greater impact on functional outcome than neurocognition [26] and it would be of interest to compare the baseline functional capacity between the clusters described in [1].

Patients in the cognitively severely impaired cluster worked for a shorter time at both follow-up data points. This is coherent with the described relation between cognitive performance and labor adaptation in schizophrenia [19]. Moreover, negative symptoms were more severe at baseline in Cluster 1 [1], and a worse quality of life has been associated with persistent negative symptoms after one year of a first episode [18]. This also seems coherent with our results, if we assume a better life quality associated with holding a paid job, although in this latter study the relation with job tenure was not specifically assessed. In treatment-resistant cases, patients with more persistent negative symptoms were less likely to continue living in the community after one year [27], also supporting a poorer quality of life associated with negative symptoms. Although neurocognition did not relate to outcome in the latter study, the corresponding cognitive examination consisted only of assessments of verbal and working memory; therefore, a complete neurocognitive examination (such as the one performed for the differentiation between Clusters 1 and 2) may be needed to properly assess that relation. The fact that at baseline Cluster 1 patients were less likely to hold a job during the first year of follow-up suggests that the relation between neurocognition and job tenure is early in this group. This, however, should be confirmed in a follow-up study in FE patients, given the inclusion of mixed chronic and FE patients in our sample.

The differences in subjective satisfaction with personal relationships between clusters were significant only at trend level, which, if confirmed, could be in the line with the relation between social cognition and interpersonal skills described in [16]. However, when directly compared Cluster 1 and Cluster 2 patients did not differ in social cognition [1]. Social cognition can be assessed using different instruments, measuring components such as emotional intelligence or social behavior, among others. These differences may contribute to those discrepancies.

The proportions of clozapine-treated were similar between these clusters, contrary to our expectations. This could indicate that treatment resistance would not be larger in Cluster 1, but clozapine prescription is highly variable even among psychiatrists in the same center [28]. The rate of clozapine prescription was similar in Cluster 1 between baseline and follow-up, but increased in Cluster 2 between those time points, suggesting an earlier severity in the former that may lead prescribers to use clozapine earlier.

Somewhat surprisingly, Cluster 2 patients showed a worse treatment compliance. Although the compliance was rather high in both clusters, even at 3rd year, that difference suggest a more frequent dropout of treatment in cases with higher functioning. A likely reason behind this are side effects of the current antipsychotics and/or, perhaps, some alleviation of symptoms associated with job tenure or, inversely, a higher need of antipsychotics where everyday life lacks its usual rewards. Although withdrawing prescriptions from the pharmacy office does not imply its intake, it can be assumed a relation between both. Direct measurements of compliance, such as plasma levels, may help in clarifying differences between clusters in this regard.

Among the limitations, our sample is relatively smaller than the one of our previous biotypes study [1] due a high dropout’s rate. Furthermore, our follow-up period was relatively short. However, our cases are well characterized from cognitive, biological and clinical points of view, and new waves of the same analyses are planned at 5th and 7th years for long-term outcomes. Besides, we did not assess at baseline everyday skills, which could influence later ability to hold job tenure. It could also be of interest to increase the sample size of bipolar patients and to control for the particular type of antipsychotics received by the patients during the follow-up. Detailed data on living environment were not collected, and duration of untreated psychosis was not available and may help clarifying the basis of outcomes differences between clusters. Finally, ours is not a study on first episodes allowing for a characterization of the outcome in the initial years of illness. Thus, the different illness duration of the cases is a potentially confounding factor that should be taken into account in the design of future studies, ideally focusing on the initial stages of the illness.

In conclusion, taken together with the biological data supporting the validity of biotypes defined on cognition, we believe that the present results give additional validity to these biotypes, which may contribute to defining possible diseases within the schizophrenia and bipolar syndromes.

## Data Availability

The dataset that support the findings of this study is available from the corresponding author upon request.
